# Concatenated convolutional neural network model for cuffless blood pressure estimation using fuzzy recurrence properties of photoplethysmogram signals

**DOI:** 10.1038/s41598-022-10244-6

**Published:** 2022-04-22

**Authors:** Ali Bahari Malayeri, Mohammad Bagher Khodabakhshi

**Affiliations:** 1 Department of Electrical Engineering, Shahid Beheshti University, 1983969411 Tehran, Iran; 2 Department of Biomedical Engineering, Hamedan University of Technology, 6516913733 Hamedan, Iran

**Keywords:** Biomedical engineering, Electrical and electronic engineering

## Abstract

Due to the importance of continuous monitoring of blood pressure (BP) in controlling hypertension, the topic of cuffless BP estimation has been widely studied in recent years. A most important approach is to explore the nonlinear mapping between the recorded peripheral signals and the BP values which is usually conducted by deep neural networks. Because of the sequence-based pseudo periodic nature of peripheral signals such as photoplethysmogram (PPG), a proper estimation model needed to be equipped with the 1-dimensional (1-D) and recurrent layers. This, in turn, limits the usage of 2-dimensional (2-D) layers adopted in convolutional neural networks (CNN) for embedding spatial information in the model. In this study, considering the advantage of chaotic approaches, the recurrence characterization of peripheral signals was taken into account by a visual 2-D representation of PPG in phase space through fuzzy recurrence plot (FRP). FRP not only provides a beneficial framework for capturing the spatial properties of input signals but also creates a reliable approach for embedding the pseudo periodic properties to the neural models without using recurrent layers. Moreover, this study proposes a novel deep neural network architecture that combines the morphological features extracted simultaneously from two upgraded 1-D and 2-D CNNs capturing the temporal and spatial dependencies of PPGs in systolic and diastolic BP estimation. The model has been fed with the 1-D PPG sequences and the corresponding 2-D FRPs from two separate routes. The performance of the proposed framework was examined on the well-known public dataset, namely, multi-parameter intelligent in Intensive Care II. Our scheme is analyzed and compared with the literature in terms of the requirements of the standards set by the British Hypertension Society (BHS) and the Association for the Advancement of Medical Instrumentation (AAMI). The proposed model met the AAMI requirements, and it achieved a grade of A as stated by the BHS standard. In addition, its mean absolute errors and standard deviation for both systolic and diastolic blood pressure estimations were considerably low, 3.05 ± 5.26 mmHg and 1.58 ± 2.6 mmHg, in turn.

## Introduction

The notion of chaos theory as a nonlinear framework in dynamical system analysis has attracted much interest. Chaotic approaches are mainly applied to emerge the complexity and nonlinear structure of the systems. Because of the nonlinear nature of biological signals, the application of chaotic framework has been found in many biological research areas, including the analysis of electroencephalograms (EEG)^1^, emotion recognition^2,3^, lung abnormality diagnosis^4,5^, and medical image processing^6^.

Cardiovascular diseases (CVD) are the leading cause of death worldwide, and hypertension is the most significant risk factor^7^. Therefore, the topic of continuous blood pressure (BP) estimation has grown increasingly in recent years. Human BPs are traditionally measured by auscultation and the oscillometric technique called the cuff-based method. In the cuff-based method, it is necessary to take significant time intervals between two successive measurements. Since the continuous measurement of BPs is vital in preventing hypertension, it cannot be applied for long-term monitoring and controlling the related CVDs^8–10^.

In long-term monitoring, the instantaneous BPs are also measured invasively through catheterization in medical centers, which restricts its appropriate usage as an ambulatory technique. Therefore, as a noninvasive and comfortable scheme, a cuffless BP estimation method based on electrocardiogram (ECG) and photoplethysmogram (PPG) has been conducted. In particular, pulse transient time (PTT) is a valuable feature for BP estimation calculated from two channels of physiological signals, such as ECG and PPG. Huynh et al.^11^ demonstrated the relationship between impedance plethysmography (IPG) and PTT to estimate BPs. A new indicator, namely, photoplethysmogram intensity ratio (PIR), is introduced by Ding et al.^12^, which can trace the low-frequency variations of BP. The results showed that the accuracy of BP estimation was improved using both PTT and PIR. Moreover, Lin et al.^13^ proposed a PTT-based approach by applying new indicators such as ascending and descending slopes, peak intensity ratio, and end valley intensity to construct BP estimation regressors. Indeed, a considerable amount of studies verified the indirect relationship between the PTT and the BP values^14^.

The indirect relationship is carried out based on the machine learning algorithms such as neural networks (NN)^15^, random forest^16^, and fuzzy systems in such PTT-based approaches^16,17^. Although such approaches require two channels of signals, there have been a number of studies that exhibit superior performance with just one channel of the physiological source. Miao et al.^7^ introduced a novel approach inspired by the efficiency of one-channel EEG signals for BP monitoring. Also, Soh et al.^18^ proposed an automated diagnostic tool for hypertension using just ECG sources. In contrast, due to the more accessible and less expensive measurement of the PPG signal, its properties have been used more widely^19–21^. However, to improve the accuracy of BP estimation, physiological parameters of both signals were eventually taken into account to determine the optimal feature vector. In particular, Esmaeilpoor et al.^22^ applied different feature sets from the PPG segments alone and in conjunction with the corresponding ECG segments. Their findings verified that the combination of both ECG and PPG waveforms contains discriminative information improving the model performance.

In recent years, deep neural networks have been widely employed in blood pressure estimation. Deep networks are inherently able to discover discriminative characteristics from input attributes without any handcrafted features. Various experiments have proved that deep networks are powerful tools in 1-dimensional (1-D) and 2-dimnesional (2-D) medical image and time-series classification problems. In this essence, convolutional neural networks (CNN) were applied to improve the accuracy of estimation. Baek et al.^23^ proposed a CNN architecture that can use raw signals for training without handcrafted feature extraction. In addition, the pure CNN architectures can be found in another group of studies for BP prediction, including Arterial blood pressure network (ABP-Net)^24^, CNN-based Siamese network^25^, visual geometry group style (VGG-style) convolutional model^26^, and spectro-temporal deep network^27^.

Due to the recurrence properties of the biological sources, the advantage of recurrent neural network (RNN) structures is also utilized in the prediction of BPs. For example, a novel multi-input multi-output dense neural network model combined with a long-short term memory (LSTM) architecture is proposed as an RNN model^28^. In the same way, stacking ANN-LSTM^29^, stacking multiple LSTM layer deep recurrent neural network (DeepRNN)^30^, and multi-stage CNN-LSTM model^22,31^ are the most recent BP estimators.

The chaotic approaches are also capable of modeling the recurrence characterization of the biological time series. In chaotic methods, the given time-series usually can be formulated in phase (state) space that leads to the introduction of nonlinear characteristics, such as Lyapunov exponents, fractal pattern feature generation^2^, and recurrence-based indices^32^. The recurrence-based algorithms provide a graphical representation of the phase space trajectory. In particular, the recurrence plot (RP) visualizes the return of the trajectory of the vicinity of its previous states in a 2-D plane^33–35^. Although RP can easily be constructed, it is sensitive to the appropriate selection of the similarity threshold^34–36^. To overcome this issue, by applying a fuzzy relation, T. D. Pham has introduced the fuzzy recurrence plot (FRP)^37–39^. In FRP, the similarity of two phase-space states is measured on the basis of fuzzy membership grades ranging between zero and one. Making use of the introduced fuzzy relation in FRP can diminish the problem of threshold selection. In addition, it was exhibited that more texture details can be visualized in FRP than that of the traditional RP^37^.

As seen, in almost all the models reviewed in the literature, the 1-D PPG and ECG sequences were applied as the network input. Therefore, 1-D convolutional layers were the more credible choice for feature generation in the network structure. In other words, these types of networks were not able to handle the 2-D convolutional layers corresponding to the 2-D input patterns. Moreover, the recursive properties and the periodicity of the input physiological signals are masked when the pure CNN models process them.

In this study, taking into account the potential ability of FRP in generating a 2-D representation of physiological signals, we updated the structure of CNNs to utilize 2-D convolutional layers. Since FRP is a recurrence-based chaotic approach, it will embed the pseudo-periodic properties of PPG signals in the prediction model, which alleviates the LSTM layers. Therefore, the 1-D PPG sequences and their corresponding 2-D FRPs are fed into the model from two separate routes, which leads to our proposed scheme, concatenated convolutional neural network (Concat_CNN). To evaluate the performance of the Concat_CNN model, we have compared it with a 1-D CNN and a 2-D CNN, which are in accordance with the PPG sequences and their FRPs, respectively. The main contributions of this paper are as follows:

- Different from the state-of-the-art studies that predict BPs through 1-D physiological signals, we examine the 2-D FRP plot as a nonlinear chaotic approach, one advantage of which is the inclusion of 2-D convolutional layers for capturing the nonlinear properties of PPG signals.
- Since FRP is constructed based on the adjacent states in phase-space, this, in turn, can include the periodicity and repetition properties of PPG in blood pressure prediction.
- Taking advantage of both sequence-based properties and recurrence-based characterization of the input PPG in the proposed Concat_CNN provides promising results.

The rest of the paper is organized as follows. In section “Fuzzy recurrence plot”, the preliminaries of the FRP are described. The proposed methodology, including the architecture of the novel deep structure, is provided in “Materials and methods”. After presenting the results, further interpretations are discussed in “Discussion” section. Finally, the paper is concluded in the last section.

### Fuzzy recurence plots

Given a time series as an output of a complex system, an RP is easily constructed by embedding the time-series into the phase space and calculation of adjacent state distances. Let $X=\{{x}_{1},{x}_{2},\dots ,{x}_{m}\}$ be an $m$-dimensional time-series data in which ${x}_{i}$ is $i$’th value of $X$. If the system trajectory is represented by $r\left(p\right), p={\rm 1,2},\dots ,N$ consisting of $N$ states, the distance between the adjacent states ${p}_{1}$ and ${p}_{2}$ are calculated as^40^:

1 $$ R_{{p_{i} ,p_{j} }} = {\varvec{\theta}}\left( {\varepsilon, \left\|r\left( {p_{i} } \right) - r\left( {p_{j} } \right)\right\|_{2} } \right);\quad p_{i} = 1,2, \ldots ,{ }N{ };\quad { }p_{j} = 1,{ }2,{ } \ldots ,{ }N $$

where $R$ is $N\times N$ recurrence matrix known as RP and ${\varvec{\uptheta}}$ is the Heaviside (step) function. When the distance between two adjacent states ${p}_{{\rm i}}$ and ${p}_{{\rm j}}$ in an RP are less than a predefined similarity threshold ($\varepsilon )$, the corresponding element in the recurrence matrix becomes one. The recurrence matrix can be visualized as a symmetric matrix by assigning black and white dots to ${R}_{{p}_{i},{p}_{{\rm j}}}=1$ and ${R}_{{p}_{i},{p}_{{\rm j}}}=0$, respectively. The main challenge in the visual representation of RPs is the appropriate selection of $\varepsilon$. In other words, in a dynamical system, the visualization of recurrence patterns in RP is significantly varied when different values of similarity threshold are considered^37^.

In the fuzzy version of RPs which are called FRP, Pham introduced a conversion of the time-series into the textural images based on a fuzzy relation measuring the similarity between two states in the reconstructed phase space^37,38^. Such fuzzy relation alleviates the challenge of threshold selection and also provides an enhancement in the visualization of the image’s textures in comparison with the conventional RPs. An FRP is also a $N\times N$ matrix whose elements are arranged to represent the distance between the states by $\mu \epsilon \{{\rm 0,1}\}$. Let $V=\{{v}_{1},{v}_{2},\dots ,{v}_{c}\}$ be the set of fuzzy clusters of the states^37^. The fuzzy membership function ($\mu$) characterizes a fuzzy relation $\mathcal{R}$ from $X$ to $V$ as a fuzzy set of $X\times V$. The fuzzy membership function with the following properties indicates the strength of the relationship of each pair of $({x}_{i}$,${v}_{k}$) in $\mathcal{R}$:

### Reflexivity

2 $$\mu \left({x}_{i},{x}_{i}\right)=1 ;\quad i={\rm 1,2},\dots ,N$$

### Symmetry

3 $$\mu \left({x}_{i},{v}_{k}\right)=\mu \left({v}_{k},{x}_{i}\right);\quad  i={\rm 1,2},\dots ,N ;\quad k={\rm 1,2},\dots ,c$$

### Transitivity

4 $$ \mu \left( {x_{i} ,x_{j} } \right) = \max\left\langle \min \left\langle \mu \left( {x_{i} ,v_{k} } \right)\right\rangle\right\rangle = \mu \left( {v_{k} ,x_{i} } \right){ };\quad { }i = j = 1,2, \ldots ,N{ };\quad k = 1,2, \ldots ,c $$

where $c$ is the number of fuzzy clusters. Herein, the fuzzy clusters of the state space are obtained by fuzzy c-means (FCM) algorithm determining the closeness between the states and the cluster centers. Subsequently, the similarity between the pairs of states is inferred using the max–min composition of a fuzzy relation. By minimizing the following objective function, the FCM algorithm categorizes the state space into $c$ overlapping clusters^37,41^.

5 $$ \begin{aligned} J\left( {U,Z} \right) & = \mathop \sum \limits_{i = 1}^{N} \mathop \sum \limits_{k = 1}^{c} \left( {\mu_{i,k} } \right)^{\omega } \left( {d\left( {x_{i} ,z_{k} } \right)} \right)^{2} \\ & \quad s.t:{ }\mathop \sum \limits_{k = 1}^{c} \mu_{i,k} ;\,\,\,i = j = 1,2, \ldots ,N{ };\quad k = 1,2, \ldots ,c \\ \end{aligned} $$

where $\omega$ is the fuzzy weighting exponent ($\omega = 2$ in this study), $Z = \left\{ {z_{1} ,z_{2} , \ldots ,z_{c} } \right\}$, $z_{k}$, and $d\left( {x_{i} ,z_{k} } \right)$ are the vector of the cluster centers, the center of the $k$th cluster, and inner-product-induced norm metric, respectively. In addition, the matrix of fuzzy $c$-partition is denoted by $U = \mu_{i,k}$. To minimize the fuzzy objective function of Eq. (5) an iterative updating process based on Eqs. (6) and (7) is applied until a stopping criterion is reached.

6 $$ \mu_{i,k} = \frac{1}{{\mathop \sum \nolimits_{j = 1}^{c} \left( {\frac{{d\left( {x_{i} ,z_{k} } \right)}}{{d\left( {x_{i} ,z_{j} } \right)}}} \right)^{{\frac{2}{\omega - 1}}} }} $$

7 $$ z_{j} = \frac{{\mathop \sum \nolimits_{i = 1}^{N} \left( {\mu_{i,j} } \right)^{\omega } x_{i} }}{{\mathop \sum \nolimits_{i = 1}^{N} \left( {\mu_{i,j} } \right)^{\omega } }} $$

**Figure 1:**
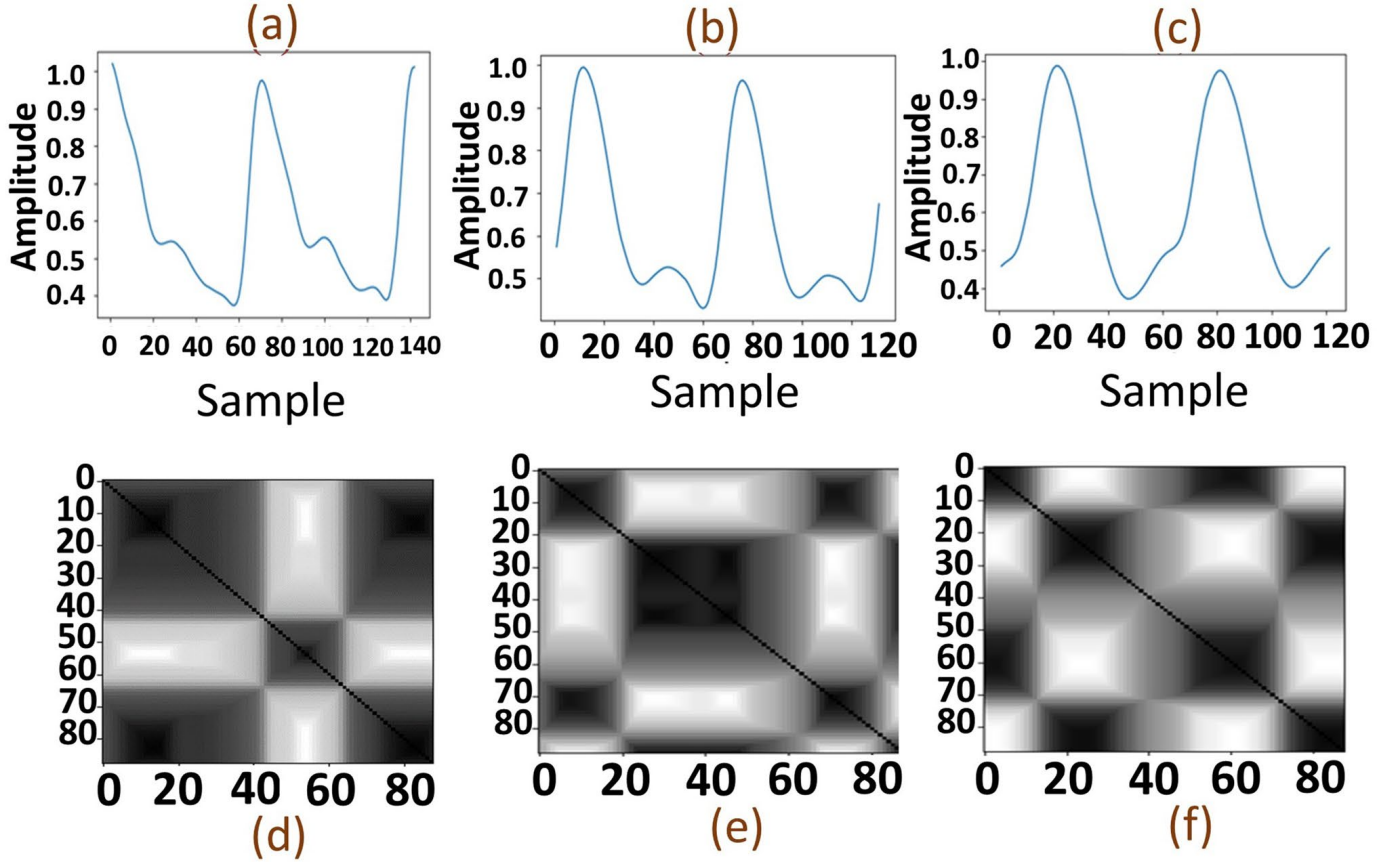
(**a**–**c**) are PPG signals and (**d**–**f**) are their respective FRPs with descending SBP and DBP at (162, 93) mmHg, (148, 74) mmHg, and (119, 52) mmHg, respectively. The recurrence properties of the PPG signals are depicted as a cluster texture features in the corresponding FRPs.

The stopping criterion is $U\left( t \right) - U\left( {t + 1} \right) \le \propto$, where $t$ is the $t$’th time step and $\propto$ is a small positive number indicating the level of accuracy. Compared with the conventional RPs, an FRP produce gray-scale images that are more advantageous than the black-white RPs^41^. Figure 1 represents the FRPs associated with the various states of the systolic and diastolic BPs. As seen, the recurrent and periodical nature of PPG signals are well represented in the corresponding FRPs.

## Materials and methods

**Figure 2:**
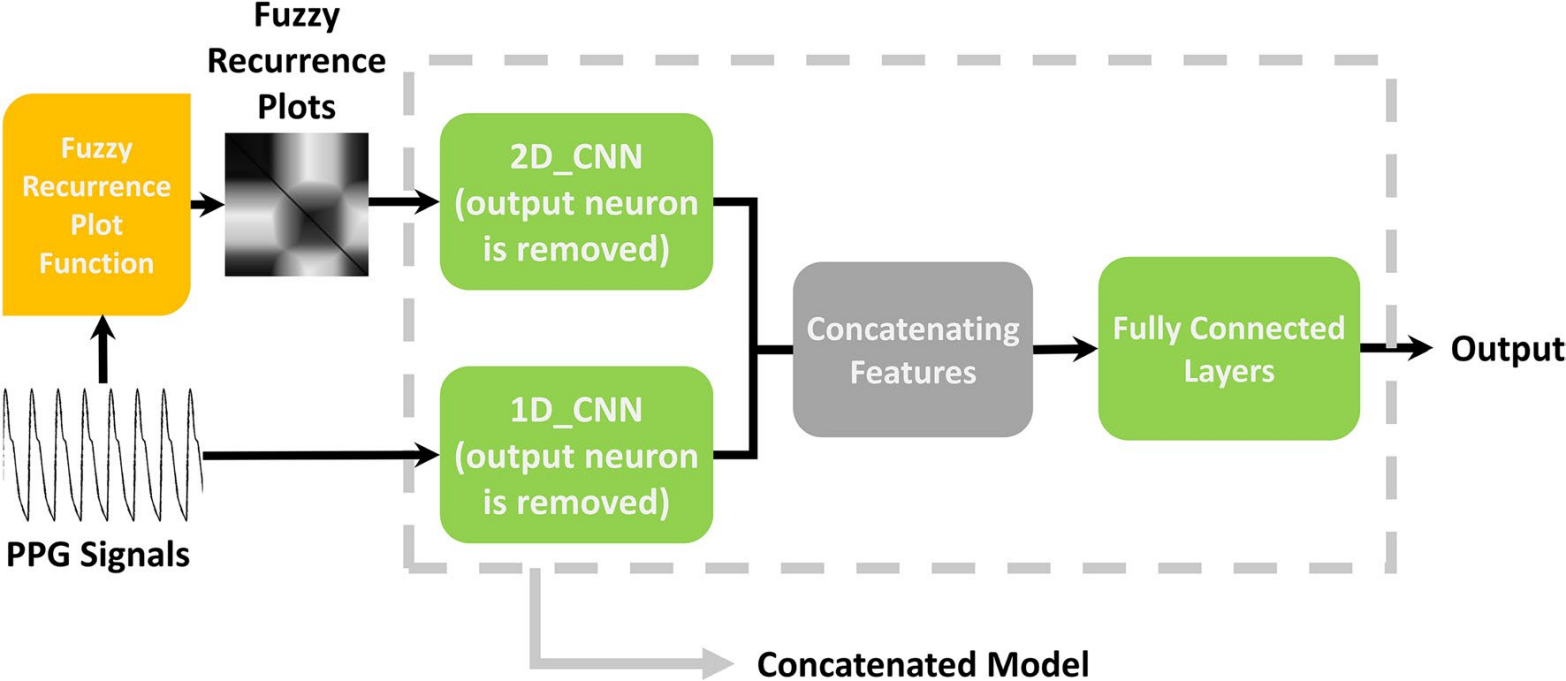
The complete preprocessing, feature extraction, concatenation and predicting stages of our proposed model.

In our method, the PPG signal is the primary input of our model. Each signal is a vector converted to a 2-D FRP, and the former signal and the later plot simultaneously are given to the model as input because our model consists of two 1D_CNN and 2D_CNN and needs two inputs, as it is depicted in Fig. 2. The 1D_CNN extracts some valuable features based on the PPG time sequences, and the 2D_CNN also does the same but is based on a 2-D FRP. After this step, both models produce a set of features that are concatenated to employ for the final decision making of the model.

### Dataset

The PPG, ECG, and invasive blood pressure of 200 patients are selected from the publically available dataset, namely, Multi-Parameter Intelligent in Intensive Care II (MIMIC-II). Data were performed in accordance with Health Insurance Portability and Accountability Act standards and the electronic records were integrated with relational database software^42^. The Institutional Review Boards of Beth Israel Deaconess Medical Center (Boston, MA) and the Massachusetts Institute of Technology (Cambridge, MA) approved the establishment of MIMIC-II^42–44^. In addition, the research had no therapeutic implication and all data were de-identified to approve patient’s confidentiality. The mean and standard deviation of ages of people who were monitored in this database were 61.6 ± 14.6 years. This database's physiological waveform records include multiple channels of simultaneously recorded signals (ECG, PPG, and arterial blood pressure), as well as time series of vital signals such as heart rate, systolic blood pressure (SBP), and diastolic blood pressure (DBP) digitized at 125 Hz. The labels (targets) for each case are SBPs and DBPs, which are acquired by an invasive blood pressure monitoring device. Since the dynamics of the input signal (PPG) and its relationship to blood pressure are changing at different times, separate processes have been used to estimate SBP and DBP.

### The proposed neural network model

CNNs are one of the first choices for researchers trying to solve complex image processing-related problems because of their outstanding results for feature learning and estimation problems. They could find the relationship between what is given to them as an input and the desired output by adjusting their layers’ weights. CNNs are formed by various layers computing convolutional transforms feeding following nonlinear and pooling operators. Researches have shown that results could be improved when fused images are used from multiple sources^45^. Also, instead of image fusion, a combination of two or even more models can result in performance enhancement.

In this study, we have proposed three different CNN networks, namely, 1D_CNN, 2D_CNN, and Concat_CNN models. 1D_CNN gets a PPG signal and predicts the BP based on the input signal, and 2D_CNN has the same operation, but its input is the 2-D plot produced by the FRP function. The third one (Concat_CNN) is a combination of two other networks. The reason we do so is that the feature maps computed in final layers for both 1D_CNN and 2D_CNN models are useful for determining blood pressure, but each of them evaluated their input from different prospects. Thus, the combination of those features before determining the output creates a type of information fusion resulting in the performance enhancement. CONCAT_CNN is a novel two-stream CNN network receiving feature maps extracted from two different 1-D and 2-D data sources as inputs. In the following sections, we first introduce the preliminaries of 1-D and 2-D CNN models utilized in this study. Then, the structure of the proposed concatenated model is presented.

### 1D_CNN

The human visual cortex was the main motivation for the invention of CNNs because they were regarded as simple computational models. They are immensely used in computer vision problems such as different sorts of detection, recognition, and augmentation. An adjusted version of 2-D CNNs, so-called 1-D CNNs, can have revolutionized the way we process 1-D biomedical signals. They remarkably eased the complexity of convolutional calculations because of the dramatically fewer number of parameters and input dimensions.

As a general finding, the majority of the researchers have used less hidden CNN layers for their 1-D CNNs architecture in comparison with 2-D CNNs. Therefore, it facilitated the process of training and implementing because of less number of unknown parameters, even fewer than 1 out of 10 times. Apart from the difference between the number of their parameters, while specific and powerful graphic processing units (GPUs) should be used to train 2-D images otherwise, it takes ages, 1-D CNNs could be trained by a normal central processing units (CPUs). Also, because of the number of parameters, 2-D CNNs require an abundance of samples to be well-trained, but even a limited labeled dataset is enough for training a 1-D CNN in most cases.

Over the forward propagation, the computation output map of a layer is the input of the following layer then this input will be convolved with the particular kernels, as bellow:

8 $$ x_{k}^{l} = b_{k}^{l} + \mathop \sum \limits_{i = 1}^{{N_{l - 1} }} conv1{\text{D}}(w_{ik}^{l - 1} .s_{i}^{l - 1} ) $$

where $s_{i}^{l - 1}$ and $w_{ik}^{l - 1} { }$ stands for the output and kernel of the *i*th neuron at layer $l - 1$, respectively, $b_{k}^{l}$ is the bias of the $k^{th}$ neuron at layer $l$, and $x_{k}^{l}$ is the input of layer $l$. Each neuron of a middle layer has an output $y_{k}^{l}$ which is computed output of the input $x_{k}^{l}$ by the activation function.

9 $${y}_{k}^{l}=f\left({x}_{k}^{l}\right){\rm  and }{s}_{k}^{l}={y}_{k}^{l}\downarrow ss$$

where $s_{k}^{l}$ is the output of the $k^{th}$ neuron of the layer $l$, and $\downarrow ss$ is the down-sampling operation of the $ss$ meaning scalar factor.

**Figure 3:**
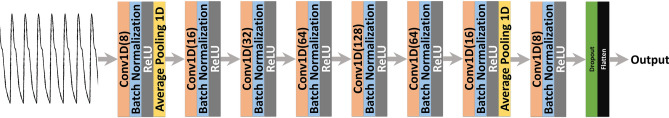
The proposed 1-D CNN network.

The architecture of our adopted 1D_CNN is depicted in Fig. 3, receiving PPG signal samples as inputs. The number of filters is mentioned in the architecture, and the kernel size of all convolutional layers is 25. Following those layers, which extracted useful features from the input signal, there are fully connected layers. Extracted feature maps are given to them to estimate desired output by passing inputs through their fully connected neurons. This model consists of 30 consecutive layers, including convolutional, batch normalization, rectified linear unit (ReLU), average pooling dropout, flatten, input, and output layers. Selecting the number of layers, the dimensions of the filters, and how they are arranged next to each other can greatly affect the performance of the network. Therefore, it is necessary to obtain a suitable model of early architecture from the models used in valid studies. To select the CNN architecture used in this study, articles that have already been done on the same database in estimating blood pressure have been considered. Specifically, in^31^, a multi-stage structure consisting of convolutional layers along with LSTM layers has been used. In the present paper, its CNN structure was considered as the initial model. However, due to the fact that this model alone does not produce a favorable result in estimating blood pressure, the modified structure is introduced in Fig. 3. This structure has been obtained by trial and error on a set of validation data. As seen, the proposed architecture consists of 1-D convolutional layer (Conv1D), batch normalization layer, ReLU activation function, average pooling, dropout, and fully connected layers.

### 2D_CNN

**Figure 4:**
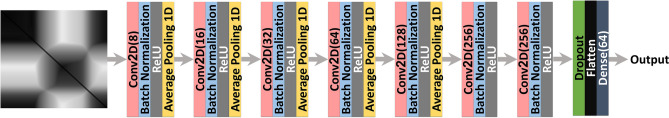
The proposed 2-D CNN network.

The second model which is proposed in our study is represented in Fig. 4. Herein, 2-D FRP images sized 89 × 89 feeding this CNN model. The processes and functions are almost similar to the previous model, but the convolution operations are 2-D instead of 1-D. As seen, there are 7 convolutional layers followed by batch normalization and ReLU layers. Next to all ReLU layers except the last two, an average pooling layer is provided to decrease the dimensionality of feature maps. There are fully connected layers to produce output which are given scalar values by those feature extractor layers. The backpropagation algorithm is used to train networks modifying layers’ weights by calculating the gradient of all parameters.

### Concat_CNN

**Figure 5:**
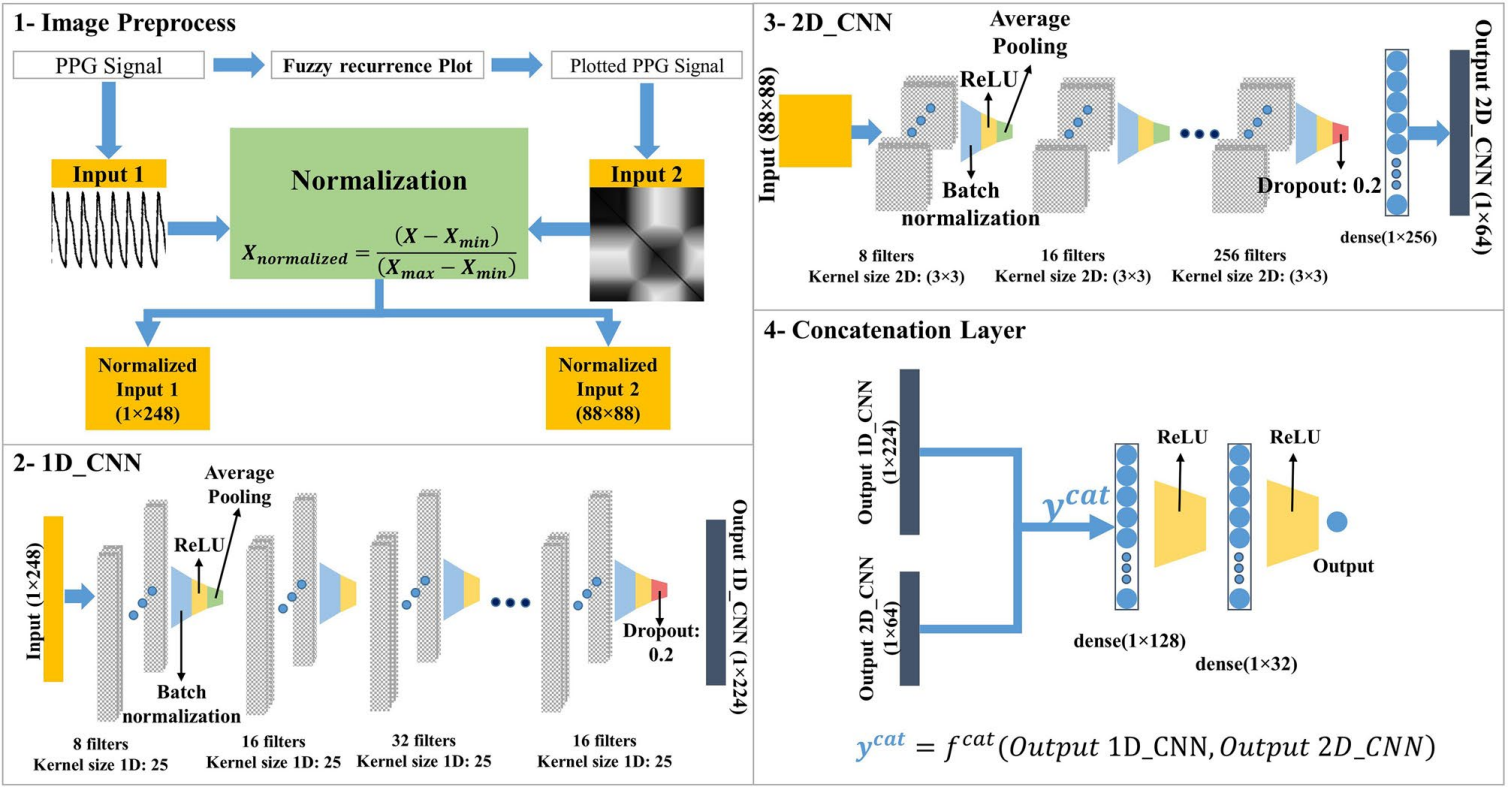
The proposed Concat_CNN network.

The main model we proposed in this paper is a mixed model from 1 and 2D_CNN introduced before. The reason why we do this is that the combined model will be equipped with the recurrence information of the input patterns without applying the formal recurrent models. Therefore, if we use that complementary information, it improves the result. In other words, the third model named Concat_CNN fuses the extracted features from the previous models, and it results in better information processing. Figure 5 represents our Concat_CNN architecture comprising parallel 1-D and 2-D CNNs followed by the concatenating layer. Indeed, convolutional streams in 1D_CNN and 2D_CNN models produce feature maps then their fusion provides the input of the fully connected layers. In the training procedure, both 1D_CNN and 2D_CNN coefficients are tuned simultaneously as a compact single network model. As seen, the proposed 2-stream network will be trained just like a single model in each epoch, a PPG segment and its corresponding 2-D FRP image fed to the Concat_CNN to modify the unknown weights with regards to their target.

There are 4 different architectures for fusing the two-stream network each of them and their formulas are investigated below. Fusion function inputs are $x_{t}^{a} \in R^{H \times W \times N}$ and $x_{t}^{b} \in R^{{H^{\prime} \times { }W^{\prime} \times { }N^{\prime}}}$ and its output is $y_{t} \in R^{{H^{\prime\prime} \times { }W^{\prime\prime} \times { }N^{\prime\prime}}}$. $H$, $W$ and $N$ represent the height, weight, and number of channels of the feature maps, in turn^45^.

### Sum fusion

$y^{sum} = f^{sum} \left( {x^{a} ,y^{b} } \right)$ add the two feature maps at same location ${ }i,{ }j$, and feature channels $n$. The computed value at point $\left( {i,{ }j,{ }n} \right)$:

10 $$ y_{i.j.n}^{sum} = X_{i.j.n}^{a} + X_{i.j.n}^{a} $$

where $$1 \le i \le H,{ }1 \le j \le W,1 \le n \le N$$ and $$X^{a} ,{ }X^{b} ,{ }y \in R^{H \times W \times N}$$.

### Max fusion

$y^{max} = f^{max} \left( {x^{a} ,y^{b} } \right)$ gives the maximum of the two input feature maps as the output:

11 $$ y_{i.j.n}^{max} = {\text{max}}\left( {X_{i.j.n}^{a} ,X_{i.j.n}^{a} } \right) $$

where $$1 \le i \le H,1 \le j \le W,1 \le n \le N$$ and $$X^{a} ,{ }X^{b} ,{ }y \in R^{H \times W \times N}$$.

### Concatenation fusion

$y^{cat} = f^{cat} \left( {x^{a} ,y^{b} } \right)$ concatenate the two input feature maps at same location:

12 $${y}_{i.j.1:n}^{sum}={X}_{i.j.n}^{a}, \quad {y}_{i.j.n+1:2n}^{sum}={X}_{i.j.n}^{b}$$

where $$y \in R^{H \times W \times 2N}$$.

### Conv fusion

$y^{conv} = f^{conv} \left( {x^{a} ,y^{b} } \right)$ initially stack two input feature maps at the same location $i,{ }j$ across the feature channels n as equations presented in the previous paragraph afterward convolves the stacked data with a bank of filters $f \in R^{1 \times 1 \times 2N \times N}$ and biases $b \in R^{D}$:

13 $$ y^{conv} = y^{cat} {*}f + b $$

where the number of output is $N$. There are a large number of parameters in deep networks, which make them complex to be trained, and it is even worse when the architecture consists of two streams. However, adding fusion layers can remarkably decrease the number of parameters in a deep network. In this paper, we employed the third method (concatenation fusion) because compared with others in this way, the number of parameters does not reduce very much. Although in some cases, reducing the number of parameters is an advantage, in our model, those features which are fused are critical for computing the output, so losing them should be avoided.

## Results

The whole steps of this experiment were carried out on Colab, which provides GPU with 12 GB of random access memory (RAM) and around 30 GB free space for loading dataset. In addition, we have used Matplotlib library in Python for drawing the figures related to the results. The dataset was 200 records chosen from MIMIC-II. They split into three training, validation, and testing parts, 70%, 10%, and 20% of each record’s length, respectively. PPG doesn’t linearly relate to blood pressure in the same way for all people because the cardiovascular dynamics of different people are varying.

**Figure 6:**
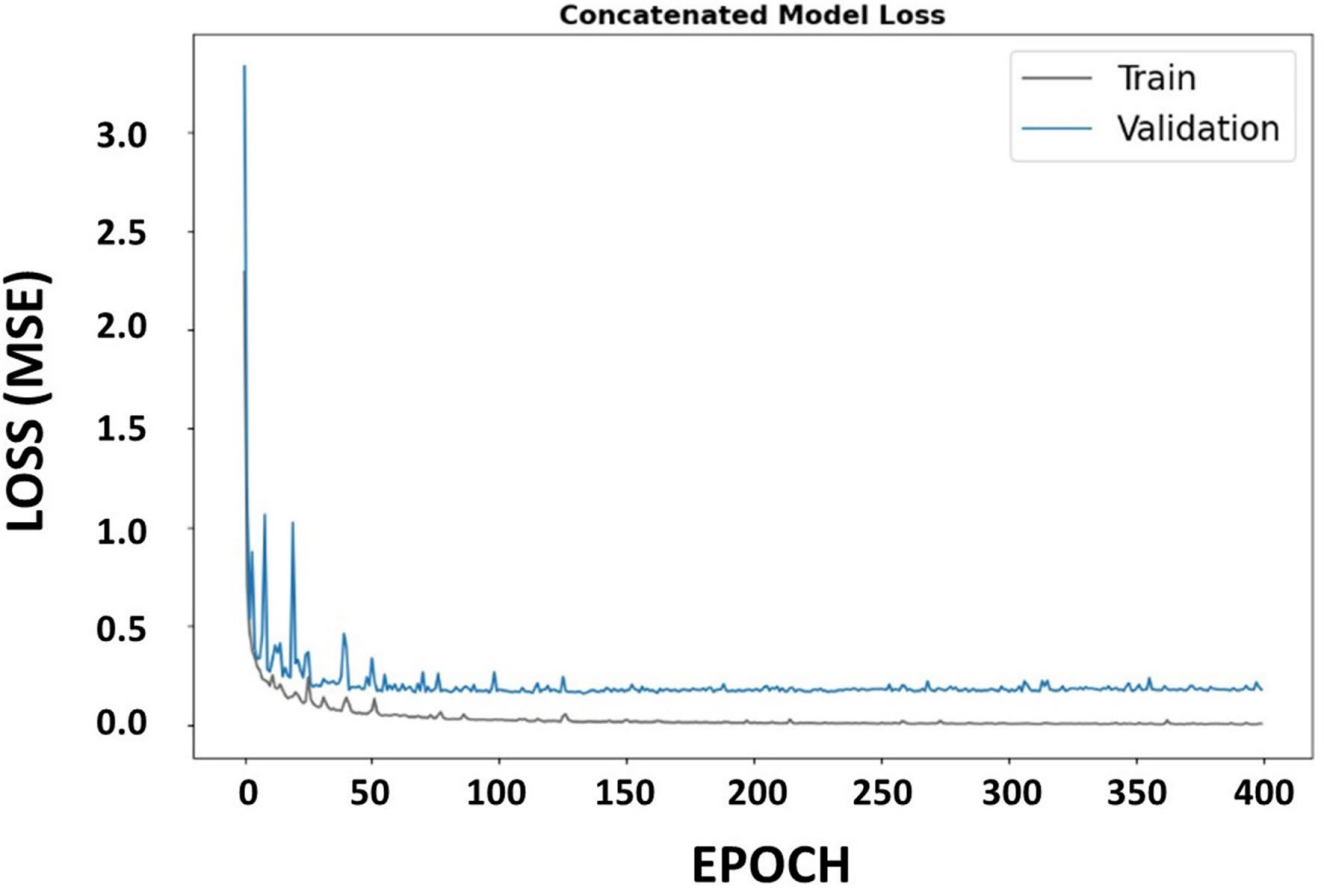
The loss graph of Concat_CNN model during training process.

Figure 6 depicts the model training error showing that the network reached maximum accuracy from 200 to 300 epochs during the training step. After that, it has started to become over fitted. All models trained with a batch size of 100 and Adam optimizer with 0.001 learning rate.

All three models were evaluated in terms of different measures, namely mean absolute error (MAE), mean squared error (MSE), and mean error (ME), for evaluation of the difference between the target and the output of each model. Moreover, the coefficient of determination (R^2^), Pearson’s correlation coefficient ($R$), and standard deviation (STD) have been calculated to determine the estimation accuracy. All formulas of the measures are presented below:

14 $$ MAE = { }\frac{1}{n}{ }\mathop \sum \limits_{i = 1}^{n} \left| {Y_{i} - \widehat{{Y_{i} }}} \right| $$

15 $$MSE= \frac{1}{n} \sum_{i=1}^{n}{({Y}_{i}-\widehat{{Y}_{i}})}^{2}$$

16 $$ ME = { }\frac{1}{n}{ }\mathop \sum \limits_{i = 1}^{n} \left( {Y_{i} - \widehat{{Y_{i} }}} \right) $$

17 $${{\rm R}}^{2}=\frac{\left[\sum_{{\rm i}=1}^{{\rm n}}{\left({{\rm Y}}_{{\rm i}}-\overline{{\rm Y} }\right)}^{2}\right]-\left[\sum_{{\rm i}=1}^{{\rm n}}{\left({{\rm Y}}_{{\rm i}}-\overline{{{\rm Y} }_{{\rm i}}}\right)}^{2}\right]}{\left[\sum_{{\rm i}=1}^{{\rm n}}{\left({{\rm Y}}_{{\rm i}}-\overline{{\rm Y} }\right)}^{2}\right]} ,\quad \overline{{\rm Y} }=\frac{1}{{\rm N}}\sum_{{\rm i}=1}^{{\rm N}}{{\rm Y}}_{{\rm i}}$$

18 $$ {\text{R}} = \frac{{\mathop \sum \nolimits_{{{\text{i}} = 1}}^{{\text{n}}} \left( {{\text{Y}}_{{\text{i}}} - {\overline{\text{Y}}}} \right)\left( {{\hat{\text{Y}}}_{{\text{i}}} - \overline{{{\hat{\text{Y}}}_{{\text{i}}} }} } \right)}}{{\sqrt {\mathop \sum \nolimits_{{{\text{i}} = 1}}^{{\text{n}}} \left( {{\text{Y}}_{{\text{i}}} - {\overline{\text{Y}}}} \right)^{2} \mathop \sum \nolimits_{{{\text{i}} = 1}}^{{\text{n}}} \left( {{\hat{\text{Y}}}_{{\text{i}}} - \overline{{{\hat{\text{Y}}}_{{\text{i}}} }} } \right)^{2} } }} $$

19 $$ STD = \sigma = { }\sqrt {\frac{{\sum \left( {Y_{i} - \overline{Y}} \right)^{2} }}{n}} $$

$Y_{i}$ is observed one which is provided in the dataset, $\widehat{{Y_{i} }}$ represents the predicted one by the model and $\overline{Y}$ is the mean of the $Y_{i}$ for $i = 1, \ldots ,n$. Also, the dataset was split into three subsets which are equal to 35,956, 10,574, and 5354 segments for training, validation, and testing, respectively.

Table 1 shows that the Concat_CNN model achieved 1.58 ± 2.60 mmHg and 3.05 ± 5.26 mmHg for $MAE$ and STD of systolic and diastolic BP, respectively. $MAE$ for this model is by around 0.2 and 0.9 mmHg lower than the other models. Note that SBP error is higher because of the fact that their values are inherently bigger than DBP, so it causes greater error but an almost equal R^2^ score.

**Table 1:** Comparison of proposed approach results presented in estimating SBP and DBP.

	Diastolic blood pressure						Systolic blood pressure					
	*R*^*2*^	*R*	*MSE* (mmHg)	*MAE* (mmHg)	*STD* (mmHg)	*ME* (mmHg)	*R*^*2*^	*R*	*MSE* (mmHg)	*MAE* (mmHg)	*STD* (mmHg)	*ME* (mmHg)
1D_CNN	0.87	0.93	9.34	2.10	2.84	− 0.26	0.87	0.93	40.53	3.92	6.35	− 0.48
2D_CNN	0.77	0.88	16.09	2.70	4.00	− 1.912	0.74	0.86	84.69	5.99	9.14	− 1.09
Concat_CNN	0.91	0.96	6.86	1.58	2.60	− 0.29	0.92	0.96	27.68	3.05	5.26	− 0.15

**Figure 7:**
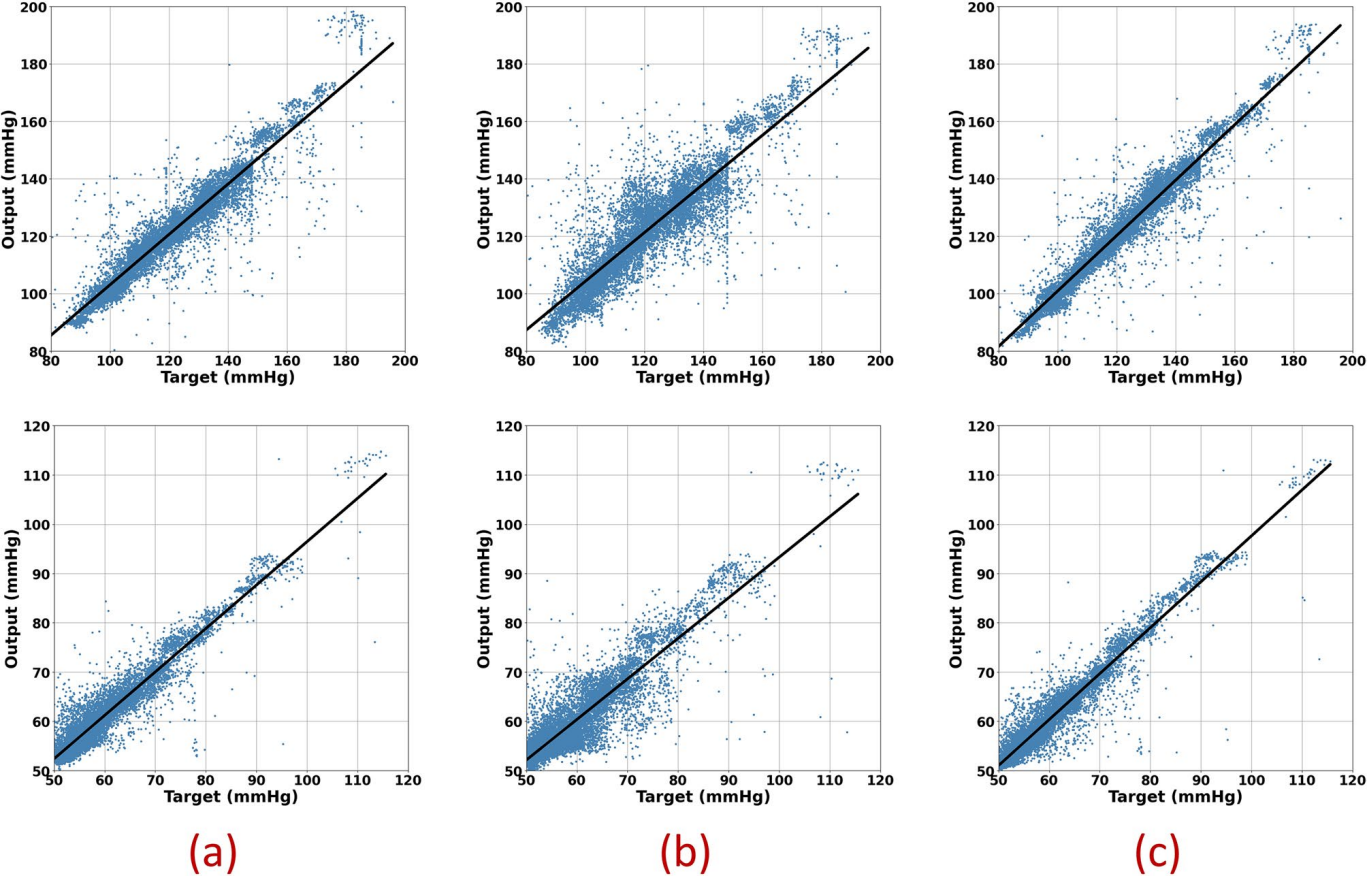
Regression plots for all 3 different models: (**a**) 1D_CNN, (**b**) 2D_CNN, (**c**) Concat_CNN. Plots in the upper row are related to systolic blood pressure and those in lower row are related to the diastolic blood pressure.

**Figure 8:**
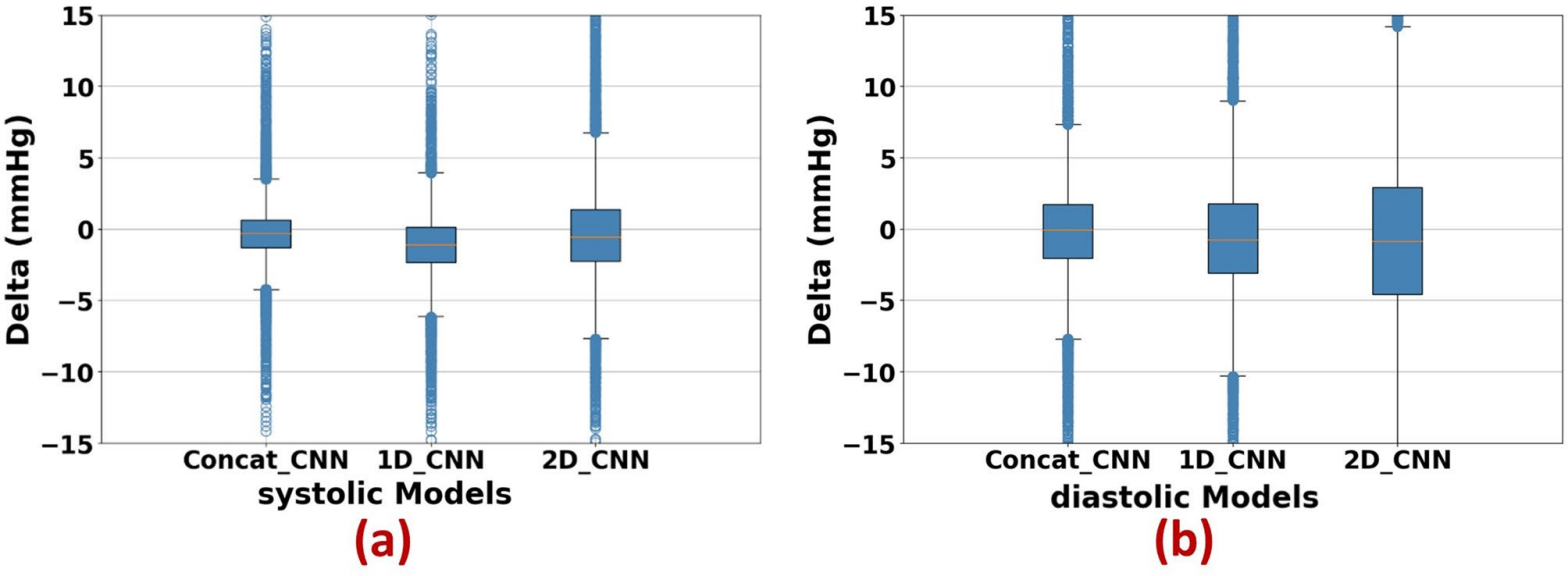
The box-and-whisker plot of estimation error for (**a**) SBP and (**b**) DBP.

**Figure 9:**
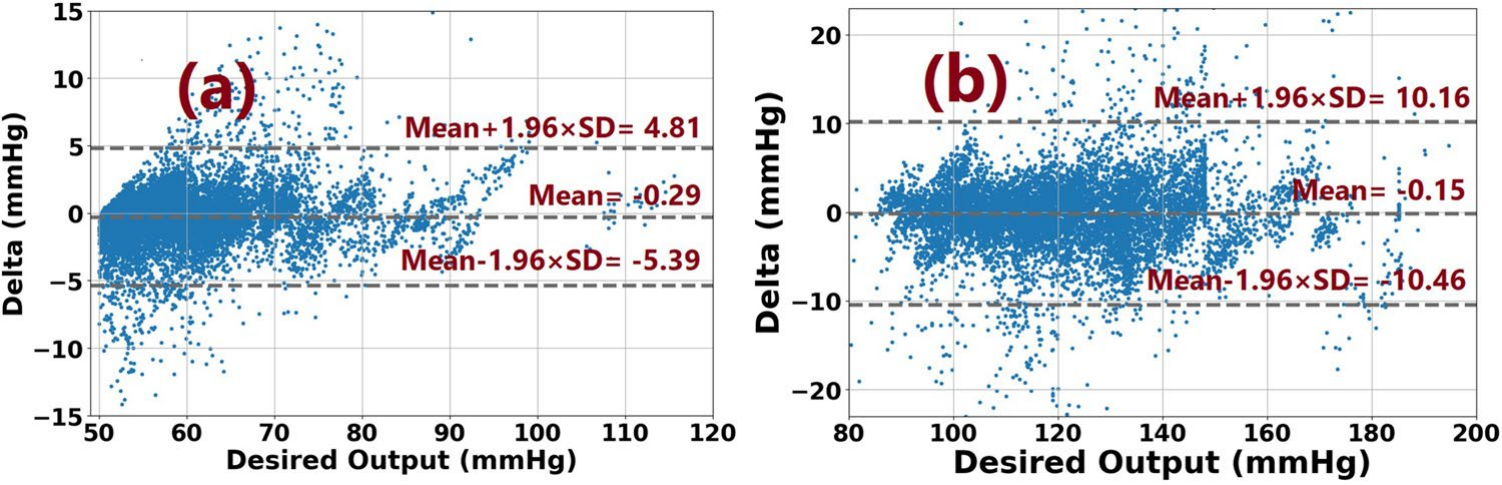
The Bland–Altman plot for DBP and SBP estimation by Concat_CNN.

Figure 7 shows regression plots for all three models, which is significantly more convergence to the targets. Figure 8 represents the box and whisker plots of the delta between the output of each model and its desired target. Both these figures illustrate the fact that the proposed two-stream concatenated model could overcome the performance of the other models. Figure 9 also demonstrates the Bland Altman diagram of systolic and diastolic blood pressure outputs of the two-stream CNN model.

Our Concat_CNN model was assessed using the British Hypertension Society (BHS) and the Association for the Advancement of Medical Instrumentation (AAMI) standard, which is presented in the next two paragraphs in detail.

### Model evaluation based on British Hypertension Society (BHS) standard

Based on the assessment criteria of the British hypertension society standard^46^, Table 2 shows the performance of the suggested method. The BHS has established standards for determining the accuracy of blood pressure monitors. Various degrees of dependability are ascribed to techniques and equipment for measuring blood pressure based on these criteria, which are determined by establishing different thresholds of error and calculating the cumulative error percentage for all estimated samples. Table 2 shows the grading system, which includes grades A, B, and C. As can be observed, if the cumulative frequency of error is less than 5 mmHg for 60% of the samples while also being greater than 85% and 95% for 10 mmHg and 15 mmHg levels, respectively, the monitoring technique is classified as grade A. Grades B and C will be given if the performance is less than the specified state (see Table 1). The approach presented in this article obtained an A grade based on the results of the measurements.

**Table 2:** Evaluation of the Concat_CNN approach based on BHS standard.

	Cumulative frequency of error	< 5 mmHg	< 10 mmHg	< 15 mmHg
BHS grades	Grade A	60%	85%	95%
	Grade B	50%	75%	90%
	Grade C	40%	65%	85%
Our Model	SBP	93.42%	97.96%	99.7%
	DBP	97.74%	99.4%	99.76

### Model evaluation based on AAMI standard

Output results were evaluated using AAMI standard, which is illustrated in Table 3. These results met all criteria because *ME* was fewer than 5 mmHg, STD was lower than 8 mmHg, and the total number of subjects was more than 85, in both diastolic BP and systolic BP. It shows the fact that this method is entirely reliable.

**Table 3:** Evaluation of the Concat_CNN approach based on AAMI standard.

Criteria of acceptance	ME of ± 5 mmHg	STD of ± 8 mmHg	Subjects > 85
Systolic blood pressure	− 0.15	5.26	200
Diastolic blood pressure	− 0.29	2.60	200

## Discussion

In general, the results verify the superiority of deep architectures in BP estimation. It improves the importance of applying nonlinear mapping between peripheral signals and blood pressure values. Previously, PPG signals or their combination with ECGs showed a strong correlation with BP^7,9,31^. However, in this study, we have just applied one source of peripheral signals in the estimation process. The PPG segments were time-series sequences made it necessary to use 1-D convolutional layers.

In addition, other studies approved the improvement of the performance of 1-D architectures when LSTM layers were adopted^29–31,47^. The results of Table 1 indicate the 1D_CNN, in turn, has yielded relatively good outcomes. In particular, in terms of R, it resulted in 0.93 for both SBP and DBP estimation. As seen, compared with the other studies, LSTM layers that model the recursive properties of the time series are of great importance for improving the results.

As a novel approach, we sought to evaluate the recursive characteristics of the PPG segments without enlarging the network structure. Therefore, instead of using LSTM layers, which create large multi-stage structures, we used a specific type of 2-D input FRP, which has the ability to model the pseudo-periodic behavior of PPG segments. Table 1 did not appear noticeable results for 2D_CNN by itself. However, the combination of 1D_CNN and the FRP could outperform the other two approaches. Specifically, in our proposed Concat_CNN model, MSE was decreased from 9.34 to 6.86 mmHg for DBP estimation and from 40.53 to 27.98 mmHg for SBP estimation. Moreover, R^2^ was increased from 0.87 to 0.91 for DBP estimation and from 0.87 to 0.92 for SBP estimation. As a result, by taking away the LSTM layers, FRP provided a 2-D image for revealing spatial and recurrence-based properties of PPG segments in BP estimation.

Figure 9 is devoted to the Bland–Altman plots for SBP and DBP. The X-axis shows the average of BPs, which are the mean of the estimations and the actual BP values. The difference between the estimated SBP and DBP and their actual values are also depicted in Y-axis. The mean of errors (bias) and the limits of agreement (${\text{bias}} \pm 1.96 \times {\text{standard deviation }}\left( {{\text{SD}}} \right)$) are exhibited in dotted lines verifying more than 95% of the points placed within the limits of agreements. This finding is consistent with the results reported in Table 3, satisfying the BHS standard may not lead to the extraction of discriminative features. Table 4 compares the results of our proposed framework with the previous studies, including traditional methods and deep architectures. The traditional methods compared here are Adaboost, random forest, and dynamical modeling. This table demonstrates that deep learning models could overcome the traditional methods in terms of R and STD. Overall, deep networks are reliable models for BP estimation because of their ability to recognize discriminative features with a huge number of parameters. Our comparative analysis also showed that the introduced framework outperformed the methods applied on the MIMIC-II dataset. The finding implies that the proposed combined model more effectively distinguishes between different conditions of BPs. In particular, the correlation coefficient (R) was 0.16, 0.08, and 0.01 greater than LSTM, Res-LSTM, and Multistage Deep Neural Network (NN), respectively, which applied LSTM layers in their structure. Therefore, the lack of LSTM employment in our proposed Concat_CNN did not cause any disruption in adding time-related information to the model. In the 2D_CNN, the size of the input FRP image may influence the approximation ability of the model. The larger the input image size, the longer the network learning time. Also, the small size of the input image may not lead to the extraction of discriminative features. This will directly affect the performance of the final Concat_CNN model. Therefore, we have applied input FRP images with different sizes of 60 × 60, 88 × 88, and 100 × 100 to the Concat_CNN, and the results are presented in Table 5. As seen, although the results are not very sensitive to image size, the lowest error values and the highest R^2^ were achieved when the FRP image size was set to 88 × 88 verifying the optimal performance.

**Table 4:** Comparison with the previous studies.

Method	Input source	DBP		SBP	
		${\varvec{R}}$	${\varvec{STD}}$(mmHg)	${\varvec{R}}$	${\varvec{STD}}$(mmHg)
PPT-Regression^12^	PPG & ECG	0.91	5.21	0.89	4.13
Dynamical approach^17^	PPG & ECG (MIMIC-II)	0.95	9.1	0.93	5.21
Random Forest^7^	ECG (MIMIC-III)	0.71	14.48	0.67	8.26
Adaboost^48^	PPG & ECG	0.59	10.09	0.48	6.14
Res-LSTM^7^	ECG (MIMIC-III)	0.88	9.99	0.71	6.29
LSTM^7^	ECG (MIMIC-III)	0.80	13.10	0.67	8.07
Multistage deep NN^31^	PPG (MIMIC-II)	0.95	5.55	0.95	2.84
ResNet^7^	ECG (MIMIC-III)	0.82	11.87	0.71	6.84
Concat_CNN	PPG & FRP (MIMIC-II)	0.96	5.26	0.96	2.60

**Table 5:** Accuracy evaluation of the different sizes of 2-D FRP images in the Concat_CNN model in terms of all metrics for DBP and SBP estimation.

Size of FRP image	DBP						SBP					
	R^2^	R	MSE (mmHg)	MAE (mmHg)	STD (mmHg)	ME (mmHg)	R^2^	R	MSE (mmHg)	MAE (mmHg)	STD (mmHg)	ME (mmHg)
100 × 100	0.89	0.94	8.77	1.85	2.87	− 0.73	0.91	0.95	30.64	3.31	5.53	+ 0.27
60 × 60	0.89	0.95	8.79	1.84	2.97	+ 0.40	0.88	0.94	38.43	3.63	6.16	− 0.73
88 × 88	0.91	0.96	6.86	1.58	2.60	− 0.29	0.92	0.96	27.68	3.05	5.26	− 0.15

Although the combination of chaotic nonlinear approaches and CNN architectures demonstrated feasible outcomes, there are some doubts while the suggested method proved its usefulness. First, the dataset was a part of the MIMIC-II database, and the results could not be fairly compared with the studies carried out on the other versions of the BP monitoring data sources. Second, we figured out that the 2D_CNN could not lonely outperform the traditional methods. Therefore, despite the use of neural networks with different architectures, employing various handcrafted features can help improve the results. Third, many varied factors like suffering from an illness, taking medicines, and so on can change the BP variations, rendering our method's results uncertain. But in the method we used, the effects of these factors were not accessible, and they had their impact on the input, and we used the same influenced data to train our model. As a potential solution, we can consider this issue as a static variable and sensitize our model to the type of participant.

Cuff-based measurement is not a proper choice for long-term monitoring of blood pressure, because of the existence of significant times between its successive BP recordings. In addition, invasive BP measurement needs catheterization, and it is not widely used for ambulatory conditions. Therefore, recent studies concentrate on the estimation of BPs through powerful soft computing approaches such as deep neural structures based on PPG and ECG. Since pseudo-periodic characteristics of PPG is of high significance in BP estimation, the usage of recurrence-based architectures is beneficial in the estimation frameworks. In this study, the recurrence properties of PPG signals are included in the model by employing their nonlinear features in phase-space. In other words, fuzzy recurrence plots are properly adopted to explore the pseudo-periodic nature of PPGs. Moreover, FRP provided 2-dimensional recurrence images to include spatial characterization of inputs to the estimation model. On the other hand, a novel double route concatenated convolutional neural network model concatenating the morphological and recurrence properties of PPGs from two separate lines is presented in our study. Our results indicated promising outcomes when sequence-based and recurrent features of PPGs were adopted in the proposed model. Although the proposed framework could overcome the methods reviewed in the literature, some disruptive factors like the subject’s illness or their medication should be considered in future studies. Also, the proposed network model could be equipped with recurrent-link architectures like long-short term memory layers compared with the pure convolutional neural network models. Since our proposed methodology concentrates on applying deep neural networks in continuous BP estimation, the gradient-based training algorithm were adopted. However, as future work our approach can be verified by the most representative metaheuristic learning-based optimization algorithms (LIOA)^49^, such as monarch butterfly optimization (MBO)^50^, earthworm optimization algorithm (EWA)^51^, elephant herding optimization (EHO)^52^, moth search (MS)^53^ algorithm, and colony predation algorithm (CPA)^54^^.^ compares the results of our proposed framework with the previous studies, including traditional methods and deep architectures. The traditional methods compared here are Adaboost, random forest, and dynamical modeling. This table demonstrates that deep learning models could overcome the traditional methods in terms of *R* and STD. Overall, deep networks are reliable models for BP estimation because of their ability to recognize discriminative features with a huge number of parameters. Our comparative analysis also showed that the introduced framework outperformed the methods applied on the MIMIC-II dataset. The finding implies that the proposed combined model more effectively distinguishes between different conditions of BPs. In particular, the correlation coefficient (R) was 0.16, 0.08, and 0.01 greater than LSTM, Res-LSTM, and Multistage Deep Neural Network (NN), respectively, which applied LSTM layers in their structure. Therefore, the lack of LSTM employment in our proposed Concat_CNN did not cause any disruption in adding time-related information to the model.

In the 2D_CNN, the size of the input FRP image may influence the approximation ability of the model. The larger the input image size, the longer the network learning time. Also, the small size of the input image may not lead to the extraction of discriminative features. This will directly affect the performance of the final Concat_CNN model. Therefore, we have applied input FRP images with different sizes of 60 × 60, 88 × 88, and 100 × 100 to the Concat_CNN, and the results are presented in Table 5. As seen, although the results are not very sensitive to image size, the lowest error values and the highest R^2^ were achieved when the FRP image size was set to 88 × 88 verifying the optimal performance.

Although the combination of chaotic nonlinear approaches and CNN architectures demonstrated feasible outcomes, there are some doubts while the suggested method proved its usefulness. First, the dataset was a part of the MIMIC-II database, and the results could not be fairly compared with the studies carried out on the other versions of the BP monitoring data sources. Second, we figured out that the 2D_CNN could not lonely outperform the traditional methods. Therefore, despite the use of neural networks with different architectures, employing various handcrafted features can help improve the results. Third, many varied factors like suffering from an illness, taking medicines, and so on can change the BP variations, rendering our method's results uncertain. But in the method we used, the effects of these factors were not accessible, and they had their impact on the input, and we used the same influenced data to train our model. As a potential solution, we can consider this issue as a static variable and sensitize our model to the type of participant.

Cuff-based measurement is not a proper choice for long-term monitoring of blood pressure, because of the existence of significant times between its successive BP recordings. In addition, invasive BP measurement needs catheterization, and it is not widely used for ambulatory conditions. Therefore, recent studies concentrate on the estimation of BPs through powerful soft computing approaches such as deep neural structures based on PPG and ECG. Since pseudo-periodic characteristics of PPG is of high significance in BP estimation, the usage of recurrence-based architectures is beneficial in the estimation frameworks. In this study, the recurrence properties of PPG signals are included in the model by employing their nonlinear features in phase-space. In other words, fuzzy recurrence plots are properly adopted to explore the pseudo-periodic nature of PPGs. Moreover, FRP provided 2-dimensional recurrence images to include spatial characterization of inputs to the estimation model. On the other hand, a novel double route concatenated convolutional neural network model concatenating the morphological and recurrence properties of PPGs from two separate lines is presented in our study. Our results indicated promising outcomes when sequence-based and recurrent features of PPGs were adopted in the proposed model. Although the proposed framework could overcome the methods reviewed in the literature, some disruptive factors like the subject’s illness or their medication should be considered in future studies. Also, the proposed network model could be equipped with recurrent-link architectures like long-short term memory layers compared with the pure convolutional neural network models. Since our proposed methodology concentrates on applying deep neural networks in continuous BP estimation, the gradient-based training algorithm were adopted. However, as future work our approach can be verified by the most representative metaheuristic learning-based optimization algorithms (LIOA)^49^, such as monarch butterfly optimization (MBO)^50^, earthworm optimization algorithm (EWA)^51^, elephant herding optimization (EHO)^52^, moth search (MS)^53^ algorithm, and colony predation algorithm (CPA)^54^.
